# The Impact of Low Influenza Immunization Rates on U.S. Hospital System Resources: A Dynamic Model Estimation

**DOI:** 10.1017/ash.2025.428

**Published:** 2025-09-24

**Authors:** Van H. Nguyen, Joaquin F. Mould-Quevedo, Andrew Rennekamp

**Affiliations:** 1 VHN Consulting, Montreal, Canada; 2 CSL Seqirus, Summit, NJ, USA

## Abstract

**Background:** Increasing influenza vaccination rates can significantly reduce the onset of severe symptoms and the risk of complications, thereby alleviating the burden on hospitals during flu seasons. However, the overall vaccine uptake has been decreasing in the United States, which is expected to increase the burden of disease. This study aims to estimate the impact of low influenza vaccination rates on disease burden and U.S. hospital system resources. **Methods:** The impact of reduced flu immunization rates was estimated using a dynamic age-stratified transmission model. Two U.S. flu seasons (2011-2012 for low incidence and 2017-2018 for high incidence) were analyzed to simulate flu epidemic variations. This study assessed four different flu vaccination rates: 25%, 30%, 35%, and 40%. Outcome measures included the number of infections, outpatient visits, hospitalizations, intensive care unit (ICU) stays, and deaths. The flu vaccine effectiveness (VE) rate was taken from CDC reports, estimating an average VE of 42% for all ages over the last 10 seasons. Vaccination rates by age group were also estimated using CDC reports, assuming immunization with quadrivalent flu vaccines for all ages. The total number of acute hospital and ICU beds available for influenza in the U.S. was assumed to be 300,000 and 30,000, respectively. **Results:** Using the U.S. flu immunization rate from the 2023-2024 season (approximately 35%), a high flu incidence season is expected to result in 71 million symptomatic infections, 29 million office visits, 0.94 million hospitalizations, and 133,670 deaths. Any scenario with an immunization rate below 45% will generate significant pressure on the U.S. hospital system and saturate the number of ICU beds during high incidence seasons. Only increasing the flu immunization rate to 50% or higher may prevent the saturation of acute hospital or ICU beds, regardless of the flu season’s incidence. **Conclusions:** The analysis shows the critical need to increase U.S. flu immunization rates to at least 50% to improve health outcomes and avoid the saturation of hospital system resources, especially ICU beds.

